# Burden of pneumocystis pneumonia in HIV-infected adults in sub-Saharan Africa: protocol for a systematic review

**DOI:** 10.1186/2046-4053-2-112

**Published:** 2013-12-12

**Authors:** Sean Wasserman, Mark E Engel, Marc Mendelson

**Affiliations:** 1 Division of Infectious Diseases and HIV Medicine, Cape Town, South Africa; 2 Department of Medicine, University of Cape Town, Cape Town, South Africa; 3 Department of Medicine, Division of Infectious Diseases and HIV Medicine, G16.68 New Main Building, Groote Schuur Hospital, Observatory, 7925 Cape Town, South Africa

## Abstract

**Background:**

Reports from Africa have suggested that pneumocystis pneumonia (PCP) is a less important cause of morbidity than in the developed world. However, more recent studies have shown high seroprevalence rates of *P. jirovecii* in healthy individuals with HIV as well as high rates of clinical disease in African children. This suggests that PCP may be more common in Africa than was previously recognised. Understanding the contribution of PCP to disease in HIV-infected individuals in sub-Saharan Africa (SSA) has important implications for diagnosis, management and resource allocation. We therefore propose to conduct a systematic review and meta-analysis in order to investigate the burden of PCP in this population.

**Methods and design:**

We plan to search electronic databases and reference lists of relevant articles published from 1995 to May 2013 using broad terms for pneumocystis, HIV/AIDS and sub-Saharan Africa. Studies will be included if they provide clear diagnostic criteria for PCP and well-defined study populations or mortality data (denominator). A novel quality score assessment tool has been developed to ensure fidelity to inclusion criteria, minimise risk of selection bias between reviewers and to assess quality of outcome ascertainment. This will be applied to eligible full-text articles. We will extract data using a standardised form and perform descriptive and quantitative analysis to assess PCP prevalence, mortality and case fatality, as well as the quality of included studies. This review protocol has been published in the PROSPERO International Prospective Register of systematic reviews, registration number CRD42013005530.

**Discussion:**

Our planned review will contribute to the diagnosis and management of community-acquired pneumonia in HIV-infected individuals in SSA by systematically assessing the burden of PCP in this population. We also describe a novel quality assessment tool that may be applied to other prevalence reviews.

## Background

Pneumocystis pneumonia (PCP), once the most common opportunistic infection in HIV in developed countries, has declined significantly since the introduction of combination antiretroviral therapy and cotrimoxazole prophylaxis in those regions [1]. In contrast, early reports from developing countries suggested that PCP was a less important cause of morbidity and mortality, and that other AIDS-associated opportunistic infections such as tuberculosis and enteric pathogens predominate [2-7]. The apparently low burden of PCP among African adults may be explained by early mortality from other more virulent infections and limited access to sensitive diagnostic tests [8,9]. This hypothesis is supported by a recent systematic review demonstrating a linear relationship between PCP diagnoses and rising gross domestic product in low- and middle-income countries [10]. It has also been suggested that environmental and virulence factors may account for the differences in incidence in Africa [11].

However, more recent studies have revealed a high seroprevalence of *P. jirovecii*, the cause of PCP, in healthy individuals with HIV [12,13] as well as high rates of clinical disease in African children [14-16]. The reported increase in prevalence among adults in the 1990s, possibly as a result of better access to diagnostics and treatment [17], may reflect the true epidemiology of PCP, suggesting that this infection may be a more important cause of community-acquired pneumonia in Africa than was previously recognised.

The World Health Organization (WHO) recommends that patients in resource-limited settings start empiric therapy for PCP on the basis of its clinical case definition [18]. A number of local guidelines have incorporated this into their management protocols. Although this approach may have an acceptable predictive value in the correct clinical and epidemiological context, inaccurate diagnosis carries a number of important implications, especially in resource-poor countries. These include incorrect assessment for intensive care unit eligibility where outcomes with PCP are reportedly poor [19] and inappropriate timing of initiation of antiretroviral therapy [20]. Over-diagnosis also exposes patients unnecessarily to the potentially toxic effects of high dose cotrimoxazole [21] and to the morbidity of delaying treatment for other diagnoses such as tuberculosis [22]. The consequences of under-treating PCP because of a misunderstanding of local prevalence are serious [11], with case fatality rates approaching 30% [10]. A better understanding of the regional prevalence of PCP would therefore support clinical management decisions and may ultimately influence outcomes.

There are a number of published reviews examining the prevalence of HIV-associated PCP in developing countries [17,22,23], but none has focused on the adult population in sub-Saharan Africa (SSA). We therefore, plan to conduct a systematic review and meta-analysis to investigate the burden of PCP among HIV-infected adults in this region, with the hypothesis that PCP has a greater contribution to morbidity and mortality than previously recognised.

### Aims and definitions

The primary outcome measures of this systematic review are the prevalence, mortality and case fatality of PCP in HIV-infected adults in SSA. We have defined prevalence as PCP diagnosed among patients with compatible symptoms and signs who would normally warrant testing at a given hospital or clinic. It does not refer to the population prevalence of disease. Mortality is defined as the proportion of deaths in a study cohort that are due to PCP (PCP deaths/total deaths), and case fatality as mortality among confirmed or probable cases (PCP deaths/total PCP diagnoses).

Secondary outcome measures include an assessment of the number and quality of studies addressing the primary research question as well as an analysis of demographic and characteristics of cases. This includes rates of co-infection with tuberculosis and other opportunistic infections. Where possible we also plan to determine the number of participants colonised with *P. jirovecii*.

### Eligibility criteria

#### Types of participants

Participants in eligible studies must reside in a SSA country at the time of enrolment and will not be excluded on the basis of legal migration status. We will analyse the data from HIV-1-infected or dual HIV-1-/2-infected adults only. We will include participants from eligible studies with an age cutoff of 15 years.

### Study design and setting

We will not limit eligibility on the basis of study design and will include autopsy studies, retrospective and prospective clinical studies. Case reports and review articles will be excluded as will any publication from a paediatric journal. All studies published before 1995 will be excluded. This date cutoff was chosen because it roughly coincides with the introduction of cotrimoxazole prophylaxis in Africa, as well as the development of improved diagnostics for PCP. There will be no restriction on the clinical setting, allowing studies analysing both in- and outpatients at any healthcare level to be included for analysis.

### Outcomes

Studies will be included if they provide clear diagnostic criteria and well-defined study populations (denominator) with mortality or case fatality data. Our definition of clinical PCP disease requires any one of the following criteria:

1. A clear clinical case definition (based upon the WHO criteria), including all of the following:

Compatible radiology: bilateral interstitial infiltrates

Compatible clinical presentation: acute/subacute, respiratory distress, hypoxia at rest or desaturation on minimal exertion

Response to PCP therapy after failing empiric antibiotics

No other explanation for presentation

2. Visualisation of oocysts/trophozoites by any method from respiratory specimen

3. Positive PCR (any method) with compatible clinical syndrome responding to PCP therapy

We have defined colonisation with *P. jirovecii* as a positive PCR (any method) on symptomatic patients who have spontaneous recovery without specific PCP therapy.

## Methods and design

### Search strategy

A broad search strategy has been designed to maximise sensitivity. This has been developed with the assistance of librarians from the Health Sciences Library at the University of Cape Town. The main search comprises individual searches using detailed medical subject heading (MeSH) terms for pneumocystis pneumonia, community-acquired pneumonia and HIV/AIDS combined with terms relevant to SSA. We plan to search Medline (accessed via PubMed), Web of Science (accessed via IST Web of Knowledge), Africa-Wide: NiPAD and CINAHL (both accessed via EBSCO Host) databases in an attempt to identify all relevant articles, irrespective of publication type, listed from 1995. There will be no language restriction. Table 1 shows an outline of our planned search strategy. We will complement this process by reviewing citations of all eligible studies and reviews identified by the search and search in Google Scholar.

**Table 1:** Search strategy

**Search**	**MeSH term (modified as needed for use in other databases)**
#1	Pneumocystis
#2	Pneumonia, pneumocystis
#3	Pneumocystis jirovecii
#4	Pneumocystis carinii
#5	#1 OR #2 OR #3 OR #4
#6	Pneumonia
#7	Community-acquired pneumonia
#8	Bronchopneumonia
#9	#6 OR #7 OR #8
#10	#5 OR #9
#11	hiv
#12	hiv infections
#13	hiv-1
#14	hiv seropositivity
#15	Aids-related opportunistic infections
#16	#11 OR #12 OR #13 OR #14 OR #15
#17	Acquired immunodeficiency syndrome
#18	Aids serodiagnosis
#19	#17 OR #18
#20	#16 OR #19
#21	Africa
#22	Africa south of the Sahara
#23	Africa, western
#24	Africa, southern
#25	Africa, eastern
#26	Africa, central
#27	#21 OR #22 OR #23 OR #24 OR #25 OR #26
#28	Angola OR Benin OR Botswana OR Burkina Faso OR Burundi OR Central African Republic OR Chad OR Congo OR Cote d Ivoire OR Democratic Republic of the Congo OR Djibouti OR Ethiopia OR Eritrea OR Equatorial Guinea OR Gabon OR Gambia OR Ghana OR Guinea OR Guinea-Bissau OR Kenya OR Lesotho OR Liberia OR Malawi OR Mali OR Mauritania OR Mozambique OR Namibia OR Niger OR Nigeria OR Rwanda OR Senegal OR Sierra Leone OR Somalia OR South Africa OR Sudan OR Swaziland OR Tanzania OR Togo OR Uganda OR United Republic of Cameroon OR Zaire OR Zambia OR Zimbabwe
#29	#27 OR #28
#30	#10 AND #20 AND #29

### Study selection

All studies retrieved by the searches will be imported into Refworks online reference management system (http://www.refworks.com). The first author will screen titles and abstracts for location, patient population and general correlation with our research objectives. Full versions of potentially relevant articles will be obtained to assess eligibility. These will then be independently evaluated for inclusion by two authors. A quality-scoring guide (described below and shown in Table 2) has been developed to allow consistent application of inclusion criteria between authors. Any disagreements on eligibility will be resolved through discussion and consultation with the third author who makes the final decision on selection.

**Table 2:** Quality assessment tool

1. Study design (selection score)	
a. Prospective clinical studies (any type)	
- Consecutive enrolment	2
- Unspecified/random enrolment	1
b. Autopsy studies	
- Consecutive enrolment	2
- Unspecified/random enrolment	1
c. Retrospective reviews (including subgroup analysis)	1
d. Review/editorial	0
e. Case report	0
2. Study objectives (selection score)	
a. Clear inclusion criteria	
- Aim related to PCP prevalence or outcomes	3
- Related to OIs/general morbidity/respiratory disease but not specific to PCP	2
- Unrelated to clinical PCP prevalence	1
b. Unclear inclusion criteria	1
3. Diagnosis^a^ (quality of outcome ascertainment)	
a. Laboratory-confirmed (any specified method/specimen)	2
b. Clinical case definition (consistent with WHO guidelines)	1
c. Not specified	0
4. Denominator	
a. Raw data denominator	2
b. Calculated denominator	1
c. No/unclear denominator/investigated < 20% of cohort for PCP/ exclusion group	0
5. Numerator (PCP numbers)	
a. Raw data numerator	2
b. Calculated numerator	1
c. No/unclear numerator (clinical PCP not mentioned/tested)	0
6. Assessment of bias (low, high or unclear risk)	
a. Attrition bias	
- Amount, nature or handling of incomplete outcome data	
b. Selection bias	
- Representativeness of the cases/cohort (clear reasons for and rates of non-inclusion)	

^a^Do not include data from clinical episodes of PCP if no case definition provided (even if study otherwise qualifies).

### Data extraction

Data will be collected independently by two individual reviewers from each eligible publication and captured onto a standardised form. We plan to extract data from text, tables and figures. Study authors will be contacted in cases of missing data or unclear eligibility criteria. The following variables will be captured:

Study characteristics

○ Year of publication and study period

○ Design, objectives and inclusion criteria

Study population

○ Country and clinical setting

○ Denominator

▪ Total recruitment/enrolment and attrition rate

▪ Numbers assessed for PCP

○ Characteristics of study participants

▪ CD4 count

▪ Exposure to antiretroviral therapy and PCP prophylaxis

Diagnostic methods

PCP prevalence

○ Definite PCP estimate with standard error of the estimate

○ Colonisation

Patient characteristics

○ Co-infections with tuberculosis and other opportunistic infections

○ CD4 count, age and gender

○ Exposure to antiretroviral therapy and PCP prophylaxis

PCP deaths

○ Mortality and case fatality

Eligible studies will be categorised according to the outcome data they provide (that is, prevalence, mortality, case fatality) and the clinical setting in which the participants are assessed. These groups are prespecified as inpatients with respiratory symptoms, unselected inpatients and all outpatients.

### Quality assessment and risk of bias

A quality assessment tool (Table 2) has been developed and will be applied to screened full-text articles in order to code eligibility decisions and to assess study quality and agreement between investigators. Our scoring system is based on the Newcastle Ottawa Scale and on a novel scale used in a previously published prevalence review [24]. It scores selection of participants with an emphasis on quality of study design; those with consecutive enrolment and transparency of inclusion criteria achieve the highest scores. A qualitative score of diagnostic methods for PCP is used to assess eligibility and quality of outcome ascertainment. There are also ratings for the quality of denominator and numerator data. Assessment of bias is built into the quality scoring scale. We plan to evaluate risk of selection and attrition bias using the Cochrane guidelines as set out in Review Manager version 5.2 (http://ims.cochrane.org/RevMan). This will inform the feasibility of and selection of studies for a pooled analysis. We also plan to conduct an assessment of the overall strength of evidence obtained from included studies. This will take into account the following factors: allocated quality and risk of bias scores described above; the total number of included studies; and consistency of results across similar clinical settings using the same diagnostic methods. Any disagreements will be resolved by discussion and consensus in consultation with the third author to resolve persistent inconsistencies.

### Data synthesis

Prevalence data from individual studies will be combined by random effects meta-analysis according to the Mantel-Haenszel method. Heterogeneity will be evaluated using the χ2-based Q statistic (significant for *P* <0.1) and the I^2^ statistic (>50% to be indicative of ‘notable’ heterogeneity [25]). STATA software version 9.2 (STATA Corporation, College Station, TX, USA) will be used to perform calculations and the meta-analysis and to produce the forest plots using the metan routine. Should standard errors (SE) not be provided, confidence intervals will be incorporated into the formula, SE = (upper limit - lower limit)/3.92.

### Presenting and reporting of results

We plan to make use of flow diagram to summarise the study selection process and detail the reasons for exclusion of studies screened as full text. This will follow the PRISMA guidelines for reporting of systematic reviews [26]. We will publish our search strategy and quality-scoring tool as supplementary documents. Primary outcome measures will be reported in the prespecified categories described above in the ‘Data extraction’ section. Quantitative data will be presented in evidence tables of individual studies as well as in summary tables and forest plots where appropriate. We plan to report on quality scores and risk of bias for each eligible study. This may be tabulated and accompanied by narrative summaries. A descriptive analysis of the strength of evidence assessment will be reported.

## Discussion

To our knowledge there are no published reports of studies that have specifically assessed the burden of PCP in SSA. We plan to address this gap with the systematic review and meta-analysis outlined in this protocol. Furthermore, there are no validated guidelines for the performance of prevalence reviews. We have developed a novel quality scoring system to enhance fidelity to inclusion criteria, reduce selection bias among investigators and to allow for a standardised assessment of the quality of included studies. This system may potentially be adapted for use in future prevalence reviews of other diseases.

The findings of this systematic review will have implications for the clinical management of HIV-associated community-acquired pneumonia in SSA. Understanding the pretest probability of PCP in this setting will help to rationalise treatment decisions and guide interpretation of diagnostic tests. This study may also inform local guidelines and management algorithms in terms of recommendations for empiric therapy and referral for further investigation. Understanding the contribution of PCP to mortality in selected populations is important for resource allocation and motivation for better diagnostic tests. Demonstration of a high case fatality may encourage more aggressive initial management and possibly earlier referral to intensive care units. Our secondary outcome measures will characterise the patients with PCP in this region and may provide a better understanding of rates of co-infection with other opportunistic pathogens. Finally, we hope to identify gaps in research that may form the basis of future studies of various aspects of pneumocystis pneumonia in SSA.

### Protocol registration

This review protocol has been published in the PROSPERO International Prospective Register of systematic reviews (http://www.crd.york.ac.uk/PROSPERO), registration number CRD42013005530.

## Competing interests

The authors declare that they have no competing interests.

## Authors’ contributions

SW conceived of the study, participated in its design and drafted the manuscript. MEE participated in study design and developed the statistical analysis plan. MM participated in design and edited the manuscript. All authors read and approved the final manuscript.
